# A Unique Case of Osteoid Osteoma Within the Mastoid Air Cell: Insights From CT Imaging

**DOI:** 10.7759/cureus.88857

**Published:** 2025-07-27

**Authors:** Ashley Cardona, Sonia Villa-Puente, Imtiaz Ahmed

**Affiliations:** 1 Radiology, Universidad Autónoma de Guadalajara School of Medicine, Guadalajara, MEX; 2 Radiology, Midwestern University Arizona College of Osteopathic Medicine, Glendale, USA; 3 Radiology, Tempe St. Luke's Hospital, Tempe, USA

**Keywords:** benign tumor, computed tomography scan, mastoid air cells, osteoid osteoma, radiologic imaging

## Abstract

Osteoid osteomas are benign, solitary bone-forming tumors associated with progressively worsening pain. The exact etiology remains unclear, although it is speculated to result from neoplasia, direct trauma, or an inflammatory process. We present a rare case of a 53-year-old male who presented to the ED with a one-year history of worsening right ear pain and no significant medical or surgical history. On physical examination, the right mastoid area was tense and swollen, without signs of inflammation. Following an enhanced and non-enhanced CT, a diagnosis of an osteoid osteoma was established. This case report contributes imaging data regarding this unusual location for an osteoid osteoma.

## Introduction

Osteoid osteomas are primary bone-forming tumors without potential for malignant transformation. Although they tend to occur in the diaphysis of long bones, their occurrence within the skull is exceptionally uncommon, with insufficient data to determine their frequency [1-5]. The incidence of osteoid osteomas is higher in males and typically occurs in individuals between the ages of 10 and 20, although cases have also been reported in older adults [1,3,5]. These tumors are typically characterized by dull aching pain, often worse at night, which is relieved by non-steroidal anti-inflammatory drugs (NSAIDs) [2]. This hallmark symptom is thought to be mediated by prostaglandin E2 production within the active nidus of the tumor [5]. Patients often experience localized tenderness and swelling, particularly if the tumor is near the surface [4].

This report describes a presentation of an osteoid osteoma within the mastoid air cells in a 53-year-old male patient with no relevant past medical history. The patient presented to the ED with a one-year history of worsening right ear pain. Physical examination revealed a tense and swollen protuberance over the right mastoid bone area. Inspection of the ear canal was unremarkable. Enhanced and non-enhanced CT scans were performed, which revealed a predominantly lucent, expansile lesion in the right mastoid air cells with an area of calcification. These findings were consistent with an osteoid osteoma, and the patient decided to continue with conservative NSAID management.

## Case presentation

A 53-year-old male presented to the ED with a one-year history of right ear pain. He reported that the pain was alleviated by aspirin and had gradually increased in intensity and persistence over the past several months. The patient denied any hearing loss, tinnitus, itching, irritation, trauma, or a history of cancer. He also reported no chronic illnesses, relevant surgical history, or use of medications or supplements. Additionally, he denied any history of illicit substance, tobacco, or alcohol use.

**Figure 1:**
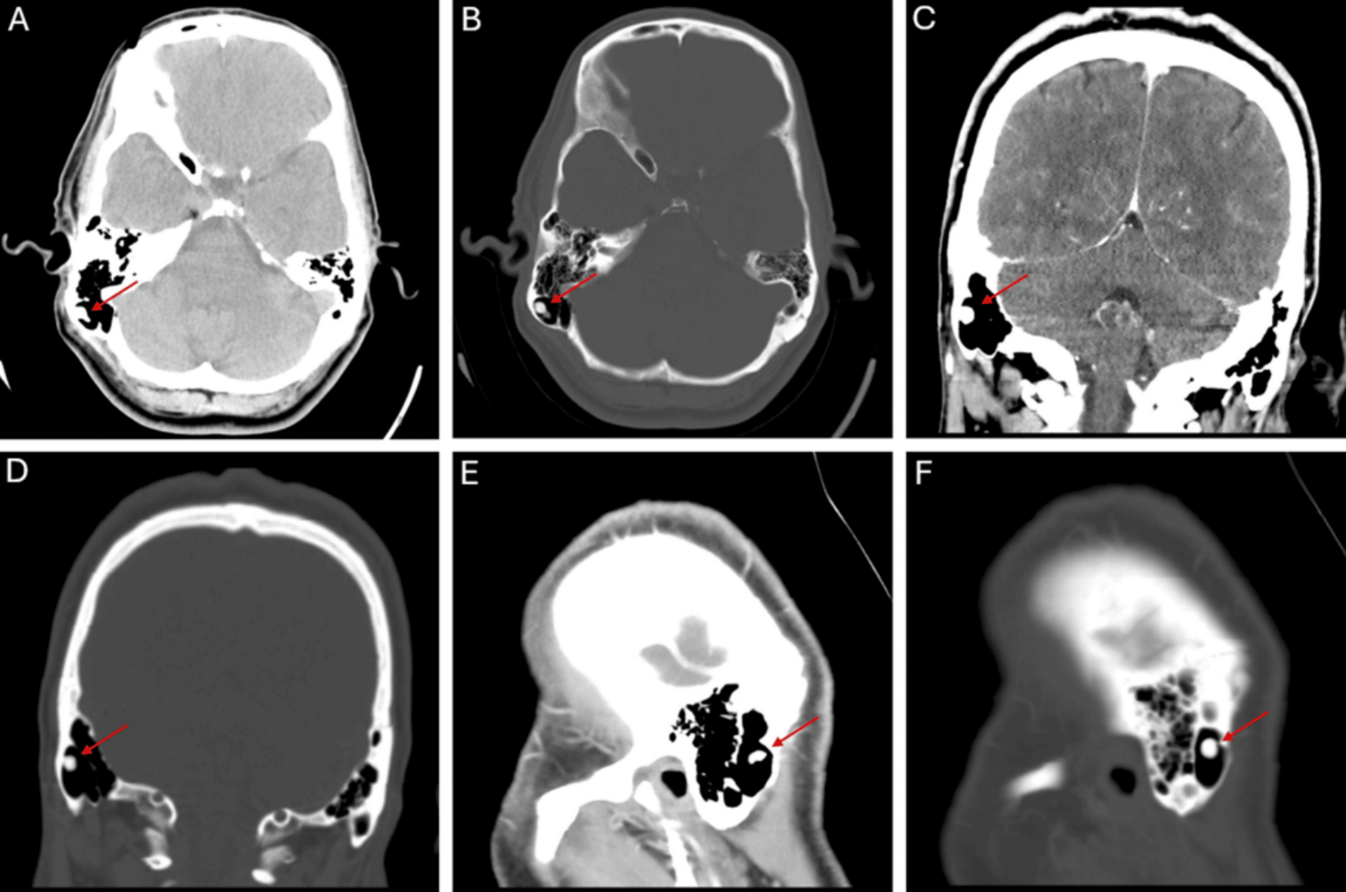
Enhanced and non-enhanced CT of the head showing a calcified nodule with a surrounding pneumatized lucent lesion in the right mastoid air cells. (A,B) Axial, (C,D) coronal, and (E,F) sagittal views are shown.

Vital signs were within normal limits, and on physical examination, the right mastoid area displayed prominent localized swelling with a palpable, non-mobile mass that had a smooth texture. The mass exhibited notable firmness, blending seamlessly with the surrounding bone, lacking distinct margins. It did not exhibit fluctuation, indicating a solid lesion rather than a fluid-filled one. There was no edema, erythema, or local warmth. Tympanic membranes were clear bilaterally without erythema, bulges, or retractions. Weber and Rinne tests were unremarkable. At this point, an enhanced and non-enhanced CT was performed, which revealed a lesion with a calcific nidus in the right mastoid air cells, as shown in Figure 1. Differential diagnoses included osteoblastoma, chronic sclerosing osteomyelitis, and Langerhans cell histiocytosis. Different treatment options were discussed, such as CT-guided radiofrequency ablation, surgical excision, and continuation of NSAIDs. The patient chose to continue with symptomatic medical management and was recommended to visit his primary care provider within one to two weeks for symptom reassessment, pain management, and referral to specialists if necessary. Repeat imaging, such as CT or MRI, in three to six months was advised to monitor the lesion, with long-term follow-up in six to 12 months to assess for any progression or need for surgical intervention.

## Discussion

Osteoid osteomas were first reported by Henry Jaffe in 1935 [6]. They are primary bone-forming tumors without potential for malignant transformation, and most commonly occur in the diaphysis of long bones, such as the femur or tibia [1,2]. Their incidence rate is approximately 2-3% of all primary bone-forming tumors and 10-14% of benign lesions [3,5]. There is an increased prevalence in males, with a ratio of 2:1-4:1, and most commonly affects individuals between 10 and 20 years of age, although cases above the age of 30 have been documented at 13% [1,3,5]. Osteoid osteomas involving the skull are exceptionally rare, and there is clear data on their frequency [3-5,7]. 

The etiology of osteoid osteomas is not fully understood, though they are speculated to result from neoplasia, trauma, or an inflammatory process [2]. They arise from aberrant osteoblastic activity and classically form a nidus composed of blood vessels, nerve fibers, interconnecting trabeculae, and unmineralized bone tissue [2,8]. These lesions are often surrounded by a ring of sclerotic bone tissue containing osteoblasts, which, when actively proliferating, produce prostaglandin E2 [2,3,8]. Pain is mediated by prostaglandin E2 and nerve fibers within the nidus, which display upregulated levels of protein S-100 [8]. The pain is typically more intense at night, often severe enough to interfere with sleep, and is relieved by NSAIDs [3].

A CT scan is the imaging modality of choice [2]. Coupled with the patient’s clinical presentation, it is sufficient to establish a diagnosis of an osteoid osteoma. On CT, they appear as well-circumscribed, low-attenuating masses with small areas of hyperdensity, measuring less than 20 mm in diameter [2,3,5]. For a lesion presenting these characteristics, the differential diagnosis may include osteoblastoma, which is typically larger than 2 cm, less responsive to NSAIDs, and may demonstrate more aggressive local behavior. Chronic sclerosing osteomyelitis should also be considered, though it usually lacks the sharply demarcated nidus of osteoid osteoma and often presents with a more diffuse inflammatory pattern. Langerhans cell histiocytosis could also be considered, as it can present with bone lesions, but it typically shows more aggressive bone destruction [1,5].

The gold standard treatment is CT-guided radiofrequency ablation, in which a probe is inserted into the target and the ablation program is initiated [5,9]. Open en bloc surgical excision has proven to be successful, although this approach carries the risk of structural instability. The resected area often involves a large margin, which can compromise bone integrity and increase the risk of fractures. If the tumor is not removed, symptoms may persist but can often be managed effectively with NSAIDs [8]. While this approach has been documented to achieve symptom resolution after 33 months, long-term NSAID use carries potential risks such as gastrointestinal irritation or renal impairment [2]. Over time, the lesion may lead to local bone remodeling and, depending on its location, it could cause limb shortening, angular deformities, or postural changes. If symptoms worsen or complications such as functional impairment arise, interventional treatment may be necessary [5]. Although rare, spontaneous regression has been reported, suggesting that observation with symptomatic management may be considered when interventional risks outweigh potential benefits or when preferred by the patient [3,9].

## Conclusions

This case report highlights the importance of considering the differential diagnosis of an osteoid osteoma in the absence of typical predisposing factors. Patient age, tumor type, lack of trauma, and location contribute to the atypical nature of this case. Use of non-enhanced and enhanced CT scans coupled with the clinical presentation is sufficient to establish a diagnosis of an osteoid osteoma. Further research is required to better understand the etiology of these tumors.
